# Targeted Delivery of Glucan Particle Encapsulated Gallium Nanoparticles Inhibits HIV Growth in Human Macrophages

**DOI:** 10.1155/2016/8520629

**Published:** 2016-11-14

**Authors:** Ernesto R. Soto, Olivia O'Connell, Fusun Dikengil, Paul J. Peters, Paul R. Clapham, Gary R. Ostroff

**Affiliations:** Program in Molecular Medicine, University of Massachusetts Medical School, 373 Plantation Street, Worcester, MA 01605, USA

## Abstract

Glucan particles (GPs) are hollow, porous 3–5 *μ*m microspheres derived from the cell walls of Baker's yeast (*Saccharomyces cerevisiae). *The 1,3-*β*-glucan outer shell provides for receptor-mediated uptake by phagocytic cells expressing *β*-glucan receptors. GPs have been used for macrophage-targeted delivery of a wide range of payloads (DNA, siRNA, protein, small molecules, and nanoparticles) encapsulated inside the hollow GPs or bound to the surface of chemically derivatized GPs. Gallium nanoparticles have been proposed as an inhibitory agent against HIV infection. Here, macrophage targeting of gallium using GPs provides for more efficient delivery of gallium and inhibition of HIV infection in macrophages compared to free gallium nanoparticles.

## 1. Introduction

Efficient targeted drug delivery systems are paramount for the development of novel and effective biopharmaceuticals and diagnostic agents. The advantages of targeted drug delivery and release include increased drug concentrations at the site of disease, limited side effects from off-target cell damage, and reduction in dose frequency and cost and length of treatment. A primary challenge for the development of drug delivery systems is the lack of optimal strategies to achieve selective and efficient cellular targeting.

Glucan particles (GPs) derived from Baker's yeast have been efficiently used for targeted payload delivery to macrophages and dendritic cells [1–11]. The hollow and porous nature of GPs (3–5 *μ*m in diameter) allows for absorption and retention of payload molecules. The *β*-1,3-D glucan surface composition also serves as an effective means of recognition by cell surface receptors in macrophages (via dectin-1 (D1) and Complement Receptor 3 (CR3)) and other phagocytic innate immune cells, allowing for a receptor-mediated particle uptake mechanism [3, 12]. We have reported several methods for efficient encapsulation of a wide range of molecules inside GPs [1–11]. Water soluble macromolecules (e.g., DNA, siRNA, and proteins) can be efficiently encapsulated inside GPs by both polyplex and layer-by-layer (LbL) synthetic approaches [1, 2]. Neutral, small drug molecules have been encapsulated by embedding the payload in polymer hydrogels [7] and encapsulation within small nanoparticles prepared* in situ* inside GPs or loaded onto the surface of GPs in a piggyback approach [6]. These different drug loading strategies accomplish two important goals: (1) high encapsulation efficiency of the payload molecule using a synthetic approach that will provide protection of the payload inside GP during storage and in transit to macrophages and (2) efficient, sustained release at low pH (pH 5 or lower) to achieve payload escape from the GPs in the endosomal compartment following macrophage uptake. Here we describe a potential new application of GPs for macrophage-targeted delivery of gallium for enhanced inhibition of HIV infection in human macrophages compared to free gallium nanoparticles.

Gallium has been shown to be effective in the treatment of several disorders, including viral and bacterial infections [13]. Gallium salts are used in pharmaceutical applications for hypercalcemia, and there is also evidence of a gallium therapeutic effect against syphilis, trypanosomiasis, and tuberculosis [14]. Gallium's therapeutic activity stems from its similarity to iron (III), with the ability to substitute for iron (III) and interfere with critical enzymatic process in metabolism and the growth of proliferating cells due to the inability of gallium (III) to be reduced in redox-active enzymes [15]. This interference is responsible for gallium inhibition of proliferation of several pathogenic microorganisms, most notably species of* Mycobacterium. *Cell uptake of gallium-transferrin complexes by infected cells, particularly macrophages, diminishes iron (III) concentration essential to enzymes likely to be involved in various processes critical for viral and bacterial infection. Additionally, gallium's lack of redox activity reduces potential toxicity effects from gallium as it does not interfere with iron (II) bearing molecules, such as heme, or with oxygen transport. Gallium is also resistant to drug efflux pumps, allowing it to be less vulnerable to drug resistance mechanisms.

Recently, gallium nanoparticles have been proposed as an inhibitory agent against coinfection with human immunodeficiency virus (HIV) and* Mycobacterium tuberculosis* (TB) [16]. Nanoparticles of gallium (III) mesotetraphenylporphyrin chloride delivered to macrophages provided a long acting effect against HIV and HIV-TB coinfection. A limitation of this system was the inefficient targeting of the proposed particles to macrophages, thus requiring large concentrations of gallium for sustained effect on HIV inhibition.

In this publication we describe an effective approach for the encapsulation of gallium compounds in the form of large nanoparticle aggregates inside GPs. This approach provides for efficient cell targeting of gallium into macrophages by receptor-mediated uptake of GP. Macrophages are a major cellular target for HIV in tissues. For example, perivascular macrophages in brain tissue are heavily infected in subjects with HIV associated dementia [17]. The selected gallium compounds are insoluble at pH 7, but they react in the acidic endosomal compartment upon GP mediated cell uptake leading to release of water soluble gallium (III). This approach represents an effective strategy for the encapsulation and macrophage-targeted delivery of gallium that could be extended to other classes of inorganic nanoparticulate materials.

## 2. Materials and Methods

### 2.1. Materials

Glucan particles were prepared from Fleishmann's Baker's yeast (AB Mauri Food Inc., Chesterfield, MO) as previously described [1]. Gallium (III) chloride (99.999% purity) was purchased from Alfa Aesar. All reagents, solvents, and buffer solutions for the syntheses and characterization of gallium particles were purchased from Sigma-Aldrich and used without further purification. Tissue culture materials were purchased from Gibco.

### 2.2. Synthesis of Gallium Nanoparticles Inside Glucan Particles (GP-Ga Formulations)

Gallium chloride was absorbed into GPs by swelling a dry GP pellet (5 mg) in a subhydrodynamic volume (5 *μ*L/mg GP) of an aqueous gallium chloride solution. GP samples containing gallium chloride were incubated at room temperature for 1 hour to allow for diffusion into the GPs. The loaded GPs were then lyophilized and the loading and lyophilization steps repeated until target concentrations of gallium chloride within the GP samples were achieved. The dry GP-GaCl_3_ pellets were then treated by wetting the pellets in a subhydrodynamic volume of trapping agent solution (sodium hydroxide, sodium bicarbonate, or sodium triphosphate) to produce insoluble gallium nanoparticles inside GPs. The GP-Ga particles were then washed with water to remove unreacted gallium chloride or small (<30 nm) gallium nanoparticles not trapped inside GPs. Samples were finally sterilized overnight in 70% ethanol and aseptically washed in 0.9% saline, and GP number was counted with a hemocytometer. Sample concentrations were then adjusted to 1 × 10^8^ particles/mL in 0.9% saline and stored frozen.

### 2.3. Characterization of Glucan Particle Encapsulated Gallium Nanoparticles (GP-Ga)

#### 2.3.1. Trapping Efficiency of Gallium in GP-Ga Formulations

Gallium was quantified using a spectrofluorometric assay [18]. Samples containing GP-Ga and empty GPs (negative controls) were suspended in 6 M HCl. Samples were centrifuged and the supernatants containing extracted gallium were mixed with rhodamine B solution in 6 M HCl (2 mg rhodamine B/mL) and incubated for 10 minutes at room temperature. The gallium-rhodamine B complexes were extracted with benzene. The fluorescence of the complexes in the organic phase was measured at an excitation wavelength of 553 nm and emission wavelength of 608 nm. GP-Ga samples incubated in aqueous rhodamine B solution were also prepared for fluorescence microscopy analysis to confirm formation of Ga-rhodamine complexes inside GPs.

#### 2.3.2. Kinetics of Gallium Release from GP-Ga

Samples containing 1 mg of GP-Ga were incubated in 1 mL of phosphate buffer saline (PBS, pH 7) or in 0.1 M sodium acetate buffer (pH 5). The samples were incubated at 37°C for a total of 4 days. At specific times, 100 *μ*L was aliquoted from samples and fresh buffer was added to maintain sink conditions. Gallium was quantified in these aliquots using the rhodamine B fluorescence assay as described above.

#### 2.3.3. GP-Ga Cytotoxicity

To determine the effect of gallium on cell growth and viability, GP-Ga samples and empty GP controls were evaluated in a B6 immortalized murine derived bone marrow macrophage (iMac) cell line [19]. Particles were evaluated for particle cell uptake at a 10 : 1 GP : cell ratio to maximize phagocytic cell uptake. Free gallium nanoparticles were also evaluated in the same concentration range as GP-Ga formulations on a gallium weight basis. Samples were suspended in complete DMEM and added to 96-well plates containing 1 × 10^4^ cells/well. The plates were incubated at 37°C under 5% CO_2_ for 24 h. Alamar blue (10 *μ*L) was added, incubated at 37°C for 30 min, and fluorescence was measured (excitation wavelength = 530 nm, emission wavelength = 590 nm). Fluorescence response is dependent on the reduction of the Alamar blue indicator by metabolically active cells and is a measure of cell number and viability. Growth arrest was calculated from the fluorescence response of the sample relative to the response of control wells containing buffer (PBS) or empty GPs.

### 2.4. HIV Infected Human Macrophage Assay to Assess Inhibition of HIV Replication

Cotransfections of 293T/17 cells with env-minus NL4.3 backbone, pHIVec2/GFP reporter, and different envelope plasmids (Env17, B33, or B59) in pSVIIIenv were done with a CaCl_2_ Promega Profection Kit (Promega Corporation, Madison, WI) [20]. Virus in supernatants was harvested 48 hours after transfection. Supernatants were collected and spun down at 1200 rpm for 5 minutes and 1 mL aliquots were made and frozen. Infectivity of each Env+ pseudovirus was estimated as GFP+ focus forming units via titration on HeLa TZM-BL cells [21].

Human monocyte derived macrophages were isolated and cultured using processed buffy coats from New York Biologics Inc. Briefly, 5 × 10^7^ Ficoll-purified peripheral blood mononuclear cells (PBMC) from a buffy coat were plated into 15 cm bacterial culture dishes for 3 h before extensively washing away nonadherent cells, culturing overnight, and repeating the washes. The adherent monocytes were then cultured for 5–7 days in 10% AB+ human plasma in DMEM. The differentiated macrophages were treated with EDTA and transferred to 48-well tissue culture dishes the day prior to infection (day −1) at 1.25 × 10^5^ cells/well. Cells were allowed to settle and adhere. After that, cells were treated with single (day −1) or multiple (days −1, 1, and 3) doses of different types of GP-Ga_2_(CO_3_)_3_ formulations, empty GPs, or free Ga_2_(CO_3_)_3_ nanoparticles. On day 0, all wells were infected with HIV Env+ pseudovirus carrying a GFP reporter gene following a laboratory standard protocol: 100 *μ*L/well (20 *μ*g/mL) DEAE dextran for 30 minutes at 37°C. HIV Env+ pseudoviruses were diluted 1 : 2 and added at 100 *μ*L/well and then spinoculated at 1200 rpm for 45 minutes. On Day 7, cells were imaged and infected GFP-positive cells were counted using a fluorescent microscope at 20x magnification. Percent infection was calculated with B33 Env+ pseudovirus alone as the 100% infected value. The assay was repeated twice to confirm trends and values. Controls were done with HIV reverse transcriptase inhibitor Zidovudine (AZT) given in multiple doses similar to the gallium formulations (20, 10, and 5 *μ*M).

## 3. Results

### 3.1. Synthesis of GP-Ga Formulations

**Figure 1:**
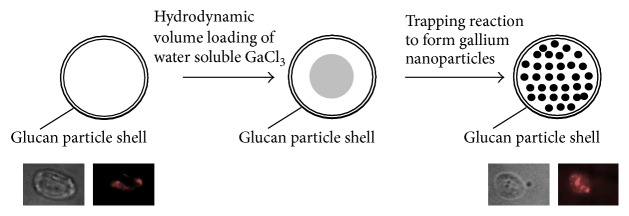
Schematic representation of gallium loading into GPs and trapping reaction to form gallium nanoparticles inside GPs. Microscopy images show complexes of rhodamine B with gallium trapped inside GPs (right image); minimum background fluorescence was observed from empty GPs (left).

A schematic representation of gallium loading and trapping inside GPs is shown in Figure 1. Dry GPs were swelled in a subhydrodynamic volume of a gallium chloride solution to load GPs by diffusion of the gallium solution into the particles through the porous structure of the glucan particle surface. The particles were then lyophilized and the loading cycle was repeated until achieving target gallium chloride concentrations inside GPs. Finally, the dry GP-GaCl_3_ particles were swollen in a subhydrodynamic volume of a solution containing a suitable reagent for a rapid displacement reaction to produce insoluble gallium oxide (Ga_2_O_3_), gallium phosphate (GaPO_4_), or gallium carbonate (Ga_2_(CO_3_)_3_) nanoparticles inside GPs. Excess reagents and small (<30 nm) insoluble gallium salts were washed from the GP samples during purification and sterilization in 70% ethanol. It is likely that the water insoluble gallium materials trapped inside GPs correspond to mixtures of compounds. For example, the reaction of gallium chloride with sodium hydroxide produces gallium oxide (Ga_2_O_3_) and it has been reported to produce other gallium compounds such as amorphous Ga(OH)_3_ and crystalline *α*-GaOOH [22]. Free gallium nanoparticles were also synthesized under the same stoichiometric conditions employed in the synthesis of gallium nanoparticles inside GPs. The objective of this work was to compare the effect of GP encapsulated gallium and free gallium nanoparticles on HIV infectivity inhibition in human macrophages. Future work will focus on complete characterization of the gallium materials in the most active GP-Ga formulation.

### 3.2. Characterization of GP-Ga Formulations

**Figure 2:**
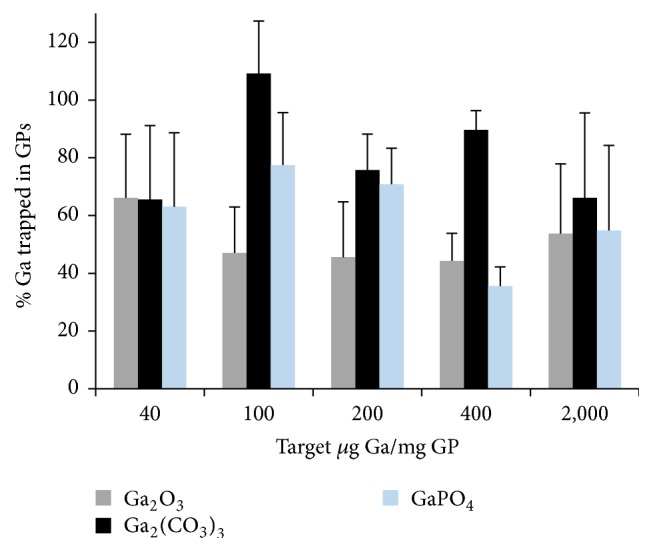
Efficiency of gallium trapping in glucan particles following reaction of gallium chloride inside GPs to yield the corresponding encapsulated water insoluble nanoparticulate gallium salt.

*Gallium Encapsulation in GPs*. The amount of gallium trapped inside GPs was quantified using a spectrofluorometric assay with rhodamine B. GP-Ga samples were digested in 6 M HCl to generate soluble gallium chloride. Gallium in acidic medium forms a stable complex with rhodamine B with higher solubility in organic solvents such as benzene. Fluorescence measurements of the gallium-rhodamine B complex extracted into benzene allowed for direct quantification of the concentrations of gallium trapped in GPs (Table 1).  Figure 2 shows these quantitative results as percentage of gallium trapped in GPs relative to the target amount of gallium chloride loaded in GPs. Gallium carbonate was trapped more efficiently (>65% trapping at all target concentrations) than gallium oxide or phosphate. Additionally, Figure 1 shows fluorescent microscopy image of a GP-Ga_2_(CO_3_)_3_ sample treated with rhodamine B confirming the trapping of the gallium salt as insoluble cores inside the hollow space of the glucan particles.

*GP-Ga Cytotoxicity*. We have previously shown that empty GPs and GPs encapsulating nanoparticles are nontoxic to cells at particle : cell ratios of up to 33 : 1 particle/cell [6]. We examined cytotoxicity of GP-Ga formulations by adding GP-Ga to the murine B6 macrophage cells at a ratio of 10 : 1 GP : cell and free Ga nanoparticles at an equivalent Ga concentration. The samples were incubated for 18–24 hours and cell viability was assessed using the Alamar blue viability assay. The results showed greater than 85% cell viability with all GP-Ga and free Ga nanoparticles formulations (not shown). 

**Figure 3:**
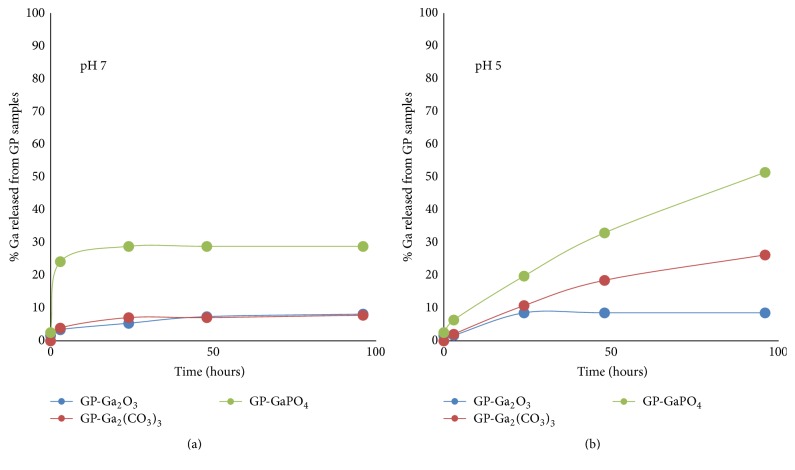
Release kinetics of gallium from GPs up to 96 hours at pH 7 (a) and pH 5 (b).

*Kinetics of Ga Release from GP-Ga Formulations*. The GP-Ga formulations were characterized for the effect of pH on kinetics of gallium release. The selection criteria for optimal GP-Ga formulations were stable formulations at pH 7 and sustained release at pH 5 (endosomal pH). Release kinetic results (Figure 3) show both GP-Ga_2_O_3_ and GP-Ga_2_(CO_3_)_3_ are highly stable at pH 7 (<10% Ga released after four days of incubation at 37°C). The GP-GaPO_4_ formulation showed a burst release of ~20% gallium and stability after the initial burst release. This burst release indicates that the gallium was not efficiently trapped as phosphate as gallium phosphate is water insoluble at pH 7. All formulations showed sustained gallium release at pH 5 with faster release for GaPO_4_, followed by Ga_2_(CO_3_)_3_, and slower release rate for Ga_2_O_3_. All samples released gallium efficiently at pH <3 (data not shown) due to a fast reaction under harsh acidic conditions to generate soluble gallium chloride.

GP-Ga_2_(CO_3_)_3_ formulations were selected for HIV infection studies based on results showing higher efficiency of Ga trapping, stability at pH 7, and better sustained release at pH 5 than GP-Ga formulations of gallium oxide or phosphate.

### 3.3. Effect of GP-Ga Treatment on HIV Infection of Human Macrophages

**Figure 4:**
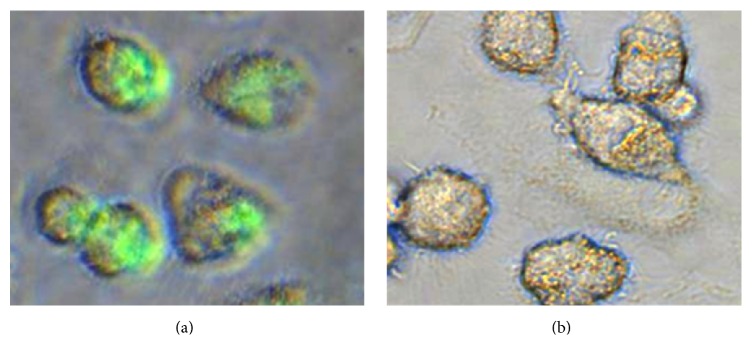
Fluorescent photomicrographs showing efficient uptake of fluorescein labeled glucan particles by human macrophages (a). No significant autofluorescence was detected from control cells (b) when exposed to UV light for same exposure time as cells treated with f-GPs.

We have previously demonstrated that GPs are effectively phagocytosed by macrophage cells via a receptor-mediated mechanism [3]. Experimental results with fluorescein labeled glucan particles using murine macrophages and dectin-1 knock-out murine macrophages demonstrated the role of the dectin-1 receptor for efficient particle uptake. The addition of a soluble beta-glucan receptor antagonist (laminarin) blocks uptake of GPs which also demonstrates that uptake occurs via a receptor-mediated mechanism. In this study, we used fluorescein labeled empty GPs (f-GP) to show that human macrophages are also capable of GP uptake. The fluorescent image in Figure 4 demonstrates efficient particle uptake by the primary human macrophages used in the HIV inhibition assays.

### 3.4. HIV Inhibition in Human Primary Macrophages with GP-Ga and Free Ga Nanoparticles

We next tested whether GP-Ga formulations could inhibit HIV infection of primary human macrophages. We selected GP-Ga_2_(CO_3_)_3_ particles to investigate, since Ga_2_(CO_3_)_3_ was trapped more efficiently compared to Ga_2_O_3_ or GaPO_4_ (Figure 2). In addition, the profiles of gallium retention at pH 7 and release at pH 5 for GP-Ga_2_(CO_3_)_3_ were superior to other formulations. Two different GP-Ga_2_(CO_3_)_3_ dosing strategies were tested for their capacity to inhibit HIV-1 pseudovirions carrying R5 macrophage-tropic B33 envelope glycoproteins (Envs) [23, 24]. Macrophages were treated with GP-Ga_2_(CO_3_)_3_ containing increasing amounts of Ga either in a single dose the day before infection (Day −1 dose) or 1 day before infection with additional doses 1 and 3 days after infection (Days −1, 1, and 3 dose). Increasing doses of GP-Ga_2_(CO_3_)_3_ increased the efficiency of inhibition, with the three-dose strategy superior to a single dose (Table 2). Inhibition reached 93–95%, although complete blockade was not observed.

**Figure 5:**
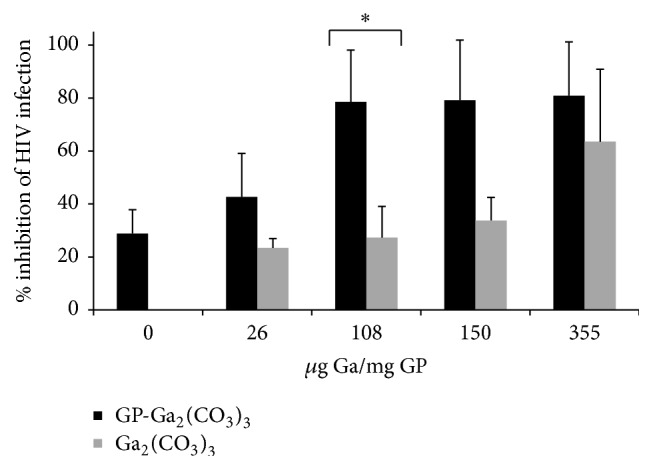
HIV infectivity inhibition in human macrophages following uptake of GP-Ga_2_(CO_3_)_3_ or free Ga_2_(CO_3_)_3_ formulations. Results are average of three experiments ± S.D. Statistical significance was determined by *t*-test; ^*∗*^ *P* < 0.01.

**Figure 6:**
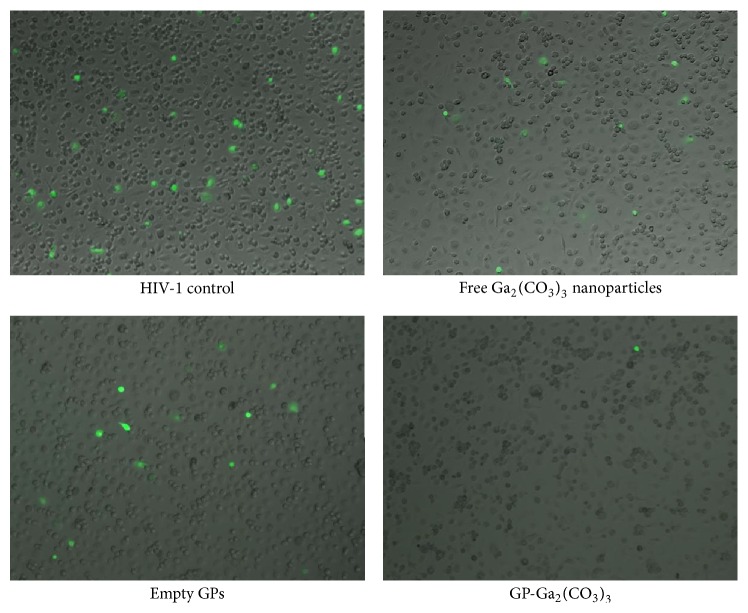
Fluorescence microscopy images showing effect of free Ga_2_(CO_3_)_3_ nanoparticles, empty GPs, and GP-Ga_2_(CO_3_)_3_ formulations on HIV growth inhibition compared to B33 HIV infected control.

We next compared GP-Ga_2_(CO_3_)_3_ to free Ga_2_(CO_3_)_3_ as nanoparticles (Ga_2_(CO_3_)_3_ NPs). It was previously reported that gallium in the form of gallium mesotetraporphyrin nanoparticles inhibited HIV infection of macrophages [16]. Figures 5 and 6 show that GP-Ga_2_(CO_3_)_3_ inhibited HIV infection of macrophages significantly more efficiently compared to free Ga_2_(CO_3_)_3_ NPs. Of note, Figure 6 indicates that empty glucan particles also inhibited infection, albeit only weakly compared to GP-Ga_2_(CO_3_)_3_.

## 4. Discussion

We show here that glucan particles can deliver gallium carbonate into primary human macrophages, where it is released over time in the low pH of endosomes to inhibit HIV-1 infection.

Glucan particles have been utilized for macrophage-targeted delivery of a wide range of payloads such as DNA, RNA, proteins, and small drug molecules [1, 2, 4–6, 11]. Gallium in the form of gallium mesotetraphenylporphyrin nanoparticles has been recently proposed as an inhibition agent for both HIV and TB in macrophages [16]. However, these gallium nanoparticles lack specificity to target macrophage cells unlike glucan particles that are efficiently targeted to macrophages by a receptor-mediated uptake mechanism. The goals of using glucan particles as carrier of gallium nanoparticles are to (1) provide receptor-mediated targeted delivery to macrophages and (2) reduce gallium dosage as uptake of a single GP provides for delivery of larger amounts of gallium than a free gallium nanoparticle.

We demonstrated efficient encapsulation of gallium in GPs by loading of water soluble gallium chloride into the particles followed by reaction with sodium salt or base to form insoluble gallium oxide, carbonate, or phosphate nanoparticles inside GPs (Figure 1). Efficient trapping of these materials requires the formation of gallium nanoparticles larger than 30 nm in diameter to prevent diffusion through the pores of the glucan particles. Gallium was encapsulated in GPs at concentrations as high as 1400 *μ*g Ga/mg GP as shown in Figure 2.

We established that glucan particles loaded with Ga_2_(CO_3_)_3_ had a superior combination of retention at pH 7 and release at pH 5 compared to other gallium salts. This experiment enabled us to devise a dosing schedule to enable Ga_2_(CO_3_)_3_ release into endosomes before and after HIV infection. Using this approach, HIV inhibition of up to 95% was achieved.

Inhibition values higher than 80% were achieved with GP-Ga_2_(CO_3_)_3_ formulations containing 108 to 355 *μ*g Ga/mg GP (Figure 5). Macrophage cells in these experiments were treated at a ratio of 10 GPs/cell in each dose for a total of 3 × 10^7^ glucan particles per 1 × 10^6^ cells for the three-day dose treatment. The gallium concentration range added to the cells increases from 6.5 *μ*g Ga/1 × 10^6^ cells for the 108 *μ*g Ga/mg GP-Ga_2_(CO_3_)_3_ formulation to 21.3 *μ*g Ga/1 × 10^6^ cells for the 355 *μ*g Ga/mg GP-Ga_2_(CO_3_)_3_ formulation. A previous study with gallium nanoparticles showed HIV inhibition following maximum uptake of 25 *μ*g Ga/1 × 10^6^ cells. However, this gallium concentration in cells was achieved by loading larger doses of gallium nanoparticles to cells. Therefore, the use of GPs provides for more efficient delivery of therapeutic doses of gallium into macrophages.

It is unclear how gallium blocks HIV infection. As discussed above, gallium's similarity to iron may enable it to interfere with critical enzyme processes during HIV entry, nuclear localization, integration, and transcription. Here, we used an HIV-1 pseudovirus carrying a GFP reporter gene controlled by the LTR promoter and expressed from an early mRNA. The inhibitory effects of gallium here could therefore be imparted at any stage during viral entry through integration and up to the expression of spliced early mRNAs.

GP-Ga_2_(CO_3_)_3_ particles therefore provide a potential delivery system to block infection of macrophages* in vivo*. Macrophages are a major target for HIV-1 infection in different tissues including the brain [17], lung [25, 26], and testes [27]. The role of macrophages during HIV transmission is less clear where CD4+ T-cells may form initial targets following sexual transmission [28]. Nevertheless, the potential of glucan particles for drug delivery at this stage in the form of microbicides would be attractive to protect against a range of sexually transmitted pathogens. Future work will address the characterization of the different phases of gallium trapped inside GPs in active formulations to correlate and optimize the effect of the gallium form with its biological activity.

## 5. Conclusions

We have developed a strategy for the encapsulation of gallium in glucan particles for targeted gallium delivery into phagocytic innate immune cells. Gallium was encapsulated in GPs in the form of gallium nanoparticles of gallium carbonate, oxide, or phosphate that are water insoluble at pH 7 providing stability and release gallium from the GPs at pH <5 (endosomal pH). GP-Ga_2_(CO_3_)_3_ formulations showed better pH 7 stability and pH 5 release profiles, so we evaluated for their activity to inhibit HIV infection in human macrophages. The GP-Ga_2_(CO_3_)_3_ formulations showed inhibition of HIV growth up to 95% and higher inhibition than free Ga_2_(CO_3_)_3_ nanoparticles. The use of GPs as carrier of Ga_2_(CO_3_)_3_ into macrophages therefore provides a potential delivery system to block HIV infection of macrophages.

## Figures and Tables

**Table 1:** Target and measured concentration of gallium trapped in glucan particles.

Target concentration *μ*g Ga/mg GP	Measured concentration *μ*g Ga/mg GP ± SD		
	Ga_2_O_3_	Ga_2_(CO_3_)_3_	GaPO_4_
40	26 ± 9	26 ± 12	25 ± 10
100	47 ± 16	108 ± 36	77 ± 18
200	90 ± 38	150 ± 28	140 ± 25
400	175 ± 38	355 ± 49	141 ± 26
2000	1063 ± 347	1308 ± 49	1085 ± 145

**Table 2:** Effect of dosage on HIV inhibition of macrophages using GPs containing gallium carbonate at different loading levels of gallium. Results are the average of three experiments. Statistical significance was determined by *t*-test, ^a^ *P* < 0.05 statistical significance between GP-Ga_2_(CO_3_)_3_ and GP control, and ^b^ *P* < 0.1 statistical significance between dosing regimes.

Sample	*µ*g Ga/mg GP	% HIV inhibition ± SD	
		Day −1 dose	Days −1, 1, 3 doses
GP	0	10 ± 9	19 ± 16
GP-Ga_2_(CO_3_)_3_	108	45 ± 2^a^	74 ± 17^a^
GP-Ga_2_(CO_3_)_3_	150	53 ± 19	92 ± 3^a^
GP-Ga_2_(CO_3_)_3_	355	44 ± 19	91 ± 2^a,b^
